# CDC’s New Healthy Aging Data Portfolio

**Published:** 2013-09-13

The CDC Healthy Aging Program has released a Healthy Aging Data Portfolio (available at http://nccd.cdc.gov/dph_aging/default.aspx ) that focuses on the health and well-being of older persons in the United States. Two factors will significantly affect health and social systems in the United States: longer adult life spans and a dramatic increase in the number of older adults, primarily because of the aging of “baby boomers” (persons born during 1946–1964). The population of U.S. residents aged ≥65 years is expected to double during the next 25 years, to about 72 million persons.

The portfolio is a compilation of previously published reports that focus on adults aged 50–64 years or ≥65 years, depending on the nature of the report. The portfolio includes the newly released report, *The State of Aging and Health in America 2013*, which provides data on key indicators and strategies to improve the health and quality of life for adults aged ≥65 years. National, state, and local public health and aging services network professionals, researchers, health-care providers, journalists, decision makers, and others interested in the health of older adults can use the portfolio to examine national, state, and selected local area data, create custom reports, learn about related expert recommendations and *Healthy People 2020* objectives, and find links to informational resources. Additional information about CDC’s work on healthy aging is available at http://www.cdc.gov/aging.

